# Neurological manifestation of Brazilian spotted fever in childhood

**DOI:** 10.1590/S1678-9946202466016

**Published:** 2024-03-18

**Authors:** Bruna Fernanda Deicke Mendes, Marina Melo Moreira, Ana Luisa Lodi Jimenez, Lívia Barbosa da Silva, Laura Maria Silva Thiersch, Carolina Malaquias Rodrigues, Bruna Ribeiro Torres, Juliana Goulart Dias da Costa, Lilian Martins Oliveira Diniz

**Affiliations:** 1 Fundação Hospitalar do Estado de Minas Gerais, Hospital Infantil João Paulo II, Belo Horizonte, Minas Gerais, Brazil; 2 Universidade Federal de Minas Gerais, Faculdade de Medicina, Belo Horizonte, Minas Gerais, Brazil; 3 Faculdade Ciências Médicas de Minas Gerais, Faculdade de Medicina, Belo Horizonte, Minas Gerais, Brazil

**Keywords:** Rickettsia infection, Brazilian spotted fever, Neurological manifestations, Tick-borne diseases, Nervous system diseases

## Abstract

Rocky Mountain Spotted Fever is a rickettsial disease caused by the bacteria *Rickettsia rickettsii*. In Brazil, the disease is known as Brazilian spotted fever (BSF), being the most significant tick-borne disease in the country. Among the affected patients, only 5% of cases occur in children aged one to nine years. Typical symptoms of the disease are fever, rash, headache and digestive symptoms. Neurological manifestations such as seizures, aphasia and hemiparesis have been described in few patients. This study aimed to describe the case of an infant diagnosed with BSF who presented severe signs of neurological manifestation.

## INTRODUCTION

Spotted fever group rickettsioses are diseases caused by an obligate intracellular Gram-negative bacteria known as *Rickettsia* and transmitted by ticks, fleas, or mites. They present a clinical spectrum ranging from a mild febrile illness to potentially fatal complications, with a 20–30% mortality rate in untreated patients^1-3^.

There are 18 species of *Rickettsia* capable of causing disease in humans^1^. *Rickettsia rickettsii* is the species that causes Rocky Mountain Spotted Fever, described in the late 19^th^ century in the United States. The disease was later described in Brazil in 1929, as Brazilian spotted fever (BSF)^3^. Despite *R. rickettsii* being the main bacterium associated to the disease, other members of the spotted fever group including *Rickettsia parkeri* have also been isolated in association with human diseases in Brazil^3^.

*Rickettsia rickettsii* infections are currently the most significant tick-borne disease in Brazil. In the country, an average of 180 cases are described per year with a mortality rate of 34%. Only 5% of cases occur in children aged one to nine years. The disease is concentrated in the Southeastern Brazil. The main vectors and reservoirs are ticks of the *Amblyomma* genus, such as *A. cajennense, A. aureolatum* and *A. ovale* which are associated with medium-sized vegetation cover, such as pastures and shrubs^3^ *. Amblyomma cajennense* complex is one of the most widely distributed tick species in the New World, and is the main vector of BSF in the Southeast and Central-West regions of Brazil^3^.

The main hosts involved in BSF disease cycle are equids, rodents, such as the capybara, marsupials, tapir, and opossums. Some mammalian hosts play crucial roles in BSF epidemiology by developing temporary rickettsemia, amplifying new generations of infected ticks, and dispersing infected vectors into the peridomiciles, such as the dogs^3^.

After a two to 14-day incubation period, the disease presents as fever, rash, headache, and digestive symptoms such as abdominal pain. Infected patients can also develop other symptoms including cough, myalgia, lymphadenopathy, nausea, vomiting, and neurological symptoms^1,4^.

The neurological manifestations of spotted fever occur in 20 to 41% of patients^5^. Headache is a typical symptom. Central nervous system (CNS) involvement is secondary to the systemic nature of the disease and the infectious vasculitis, but direct invasion has also been documented^1,4,5^. Convulsions and sensory alteration have also been described; although, more severe manifestations such as hemiparesis, cranial nerve paralysis or coma are quite rare^4-7^.

Few cases of neurological involvement in children with spotted fevers had been described in India, Mexico, and the United States^4-6^. Early recognition and treatment of the infection are important to prevent the morbidity and mortality associated with these manifestations^1^. This study aimed to describe the case of an infant diagnosed with BSF in Minas Gerais State, Brazil, who presented severe signs of neurological manifestation.

## CASE REPORT

Male patient, 1 year and 8 months old, residing in a rural area, sought emergency care with a history of fever for six days and rash on the trunk, limbs, and face. Four days after the onset of the condition, he stopped walking and speaking. The mother reported that she had found a tick on the child’s body about 15 days before fever had begun.

**Figure 1:**
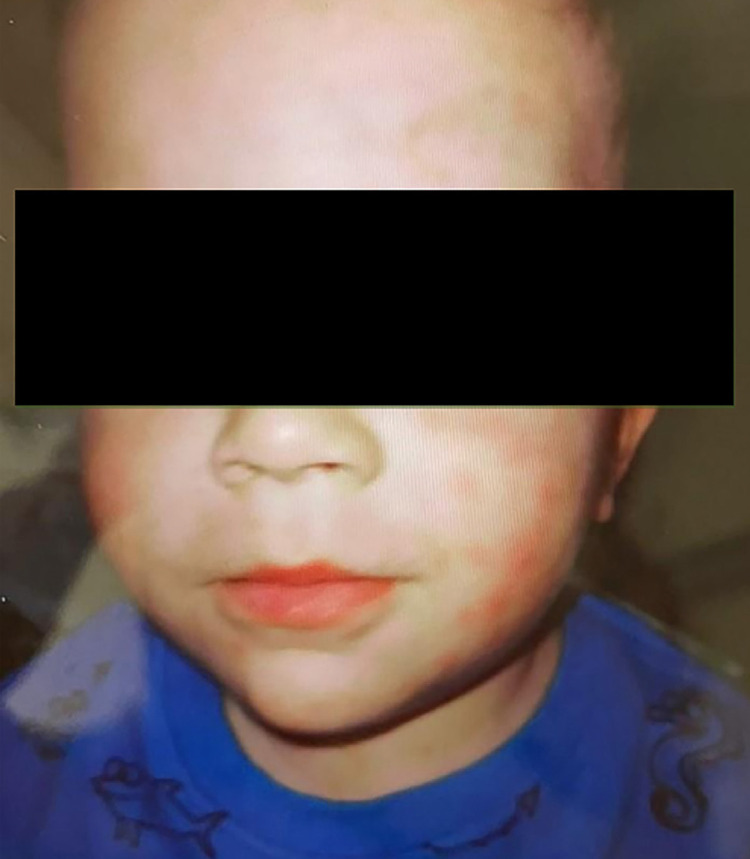
Presence of maculopapular rash on the face.

**Figure 2:**
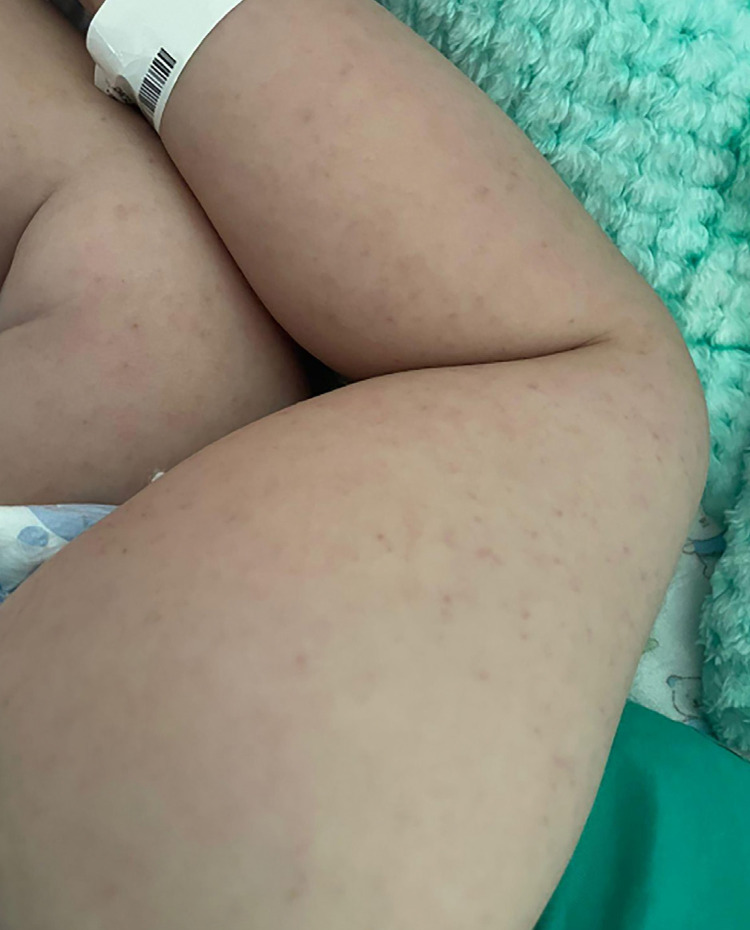
- Petechial rash on the limbs.

Upon hospital admission, petechial rash on the limbs, neck stiffness, vision loss, aphasia, and left hemiparesis were observed. (Figures 1 and 2) Given the clinical and epidemiological data, the hypotheses of meningococcal disease and BSF were considered and intravenous ceftriaxone and oral doxycycline were immediately administered. Laboratory tests showed anemia (Hb = 8.9 mg/dL), thrombocytopenia (70,000/mm^3^), C-reactive protein 30 mg/dL, and hyponatremia (134 mg/dL). A cerebrospinal fluid (CSF) analysis was performed showing 80 cells/mm^3^ (88% lymphocytes), glucose = 43 mg/dL and proteins=95 g/L. Cranial computed tomography on admission was normal. Dengue rapid test (NS1) was performed showing negative result. Polymerase chain reaction (PCR) test and indirect immunofluorescence reaction for BSF were collected upon hospital admission and sent to reference laboratory of Minas Gerais State.

The fever stopped after the third day of treatment, however, due to the neurological symptom persistence, the patient was transferred to a reference hospital in the state capital, for continuity of care. On admission, nine days had passed since the onset of symptoms, and despite no longer having fever, the patient still showed significant sensory oscillation ranging from irritability to drowsiness, making poor eye contact, aphasic, with neck stiffness and a positive Brudzinski’s sign, showing significant axial hypotonia. Ceftriaxone and doxycycline were continued, and intravenous acyclovir was administered due to the suspicion of herpetic encephalitis. On the fourth day of treatment a new CSF analysis was performed, showing a slight improvement: 10 cells/mm^3^ (68% lymphocytes), glucose = 48 mg/dL and proteins = 36 g/L. On the same day, CSF fluid was also tested for *Rickettsia* spp., herpesvirus, dengue virus, chikungunya virus, meningococcus and pneumococcus, by polymerase chain reaction (PCR) assay. Serum samples were also collected for a new BSF serologic test. New laboratory tests showed improvement in anemia status (Hb=10mg/dL) and thrombocytopenia (463,000/mm^3^), leukocytosis (19,760 cel/mm^3^), increased CRP (97.9 mg/dL), and maintenance of hyponatremia (134 mg/dL).

On the fifth day of treatment, the patient showed improvement in eye contact, following movements with his gaze, still maintaining neck stiffness, aphasia and axial hypotonia. On the same day, PCR test for *Rickettsia* spp., performed upon admission to the hospital, on the sixth day since onset of symptoms, was made available showing a positive result, corresponding to the presence of a specific band (434 bp) and to the 17 Kda antigen fragment amplicon (rickettsiosis). Serologic exams (indirect immunofluorescence reaction) for BSF, that were performed on the same day, resulted negative for IgM and IgG. All other PCR tests were negative.

Ceftriaxone and acyclovir were stopped after five days and doxycycline was maintained for 10 days. On the eighth day of treatment, the patient was able to walk and talk again, showing complete resolution of the neurological symptoms. The second serologic exam for BSF carried out 10 days after the beginning of fever and neurological symptoms showed positive results (IgM = 1/512 and IgG = 1/128).

The child was discharged after 10 days of treatment. Then, after 14 days of outpatient follow-up, the child had recovered completely and no longer had any neurological symptoms.

## DISCUSSION

Infections caused by *Rickettsia* constitute an area of great interest in public health due to their impact on the population^8^. In Brazil, most cases of BSF occur from June to October. In 2023, until the end of June, Brazil had already registered 49 cases of BSF and four deaths^9^.

Only 3–18% of children present the typical picture of the disease, which makes its diagnosis difficult during the first days of infection^1,4,5^. In addition, the rash with a maculopapular or petechial appearance, which occurs in most children, can be confused with other exanthematous febrile illnesses of childhood, such as meningococcal disease, dengue fever, and mononucleosis^2^. Laboratory abnormalities such as thrombocytopenia, anemia, and hyponatremia can help recognize the disease^2^.

The neurological involvement in *Rickettsia* diseases occur in children younger than 10 years and few cases are described in infants^1,4,6^. The diagnosis should be considered in cases of aseptic meningitis, encephalitis, and acute disseminated encephalomyelitis, in patients living in risk areas^5^. The most common neurological manifestations are headache (44–90%), irritability (37–61%), sensory alteration (23–56%), and seizures (17–59%)^6^. Focal deficits, as observed in our patient, are described in less than 15% of the cases^4,5^. A Brazilian study including 44 children diagnosed with BSF identified that only 10% had neurological symptoms such as seizures and coma. Hemiparesis, aphasia, and visual loss, as described in our patient, were not observed^10^. A study carried out in India with 51 patients identified hemiparesis as a symptom present in only 2% of cases^4,5^. Deafness, speech disorders, and visual loss have been rarely described^1^.

The presence of meningeal signs as observed in the present case ranges from 16–37%. CSF abnormalities are observed in 76% of patients subjected to CSF puncture, with slightly elevated proteins, mild pleocytosis with lymphocytic or polymorphonuclear preponderance and normal glycorrhachia, making the differential diagnosis with infectious diseases of the CNS a great challenge^3,4,5,7^. Various neuroimaging abnormalities have been described, such as cerebral edema, meningeal enhancement, blurring of sulci, cerebral infarction, and increased intracranial pressure^5^. In a few cases, the image may be normal, as observed in our patient^4,5^.

Indirect immunofluorescence reaction is the most used serological method, despite the antibodies are detectable only between the 7^th^ and 10^th^ day of infection, hampering early diagnosis. Our patient had an initially negative test upon hospital admission, and its positivity was observed only when it was repeated a few days later. In addition, molecular biology techniques (PCR) have been made available to facilitate the diagnosis, despite their low sensitivity^3^. PCR-based amplification methods are useful rickettsial diagnostic tools in the early phase of the illness and detects species from the spotted fever group. In our case, PCR assay was essential for early diagnosis.

Our patient initially received treatment for bacterial meningitis, viral encephalitis, and BSF, since the initial clinical picture did not allow for the differentiation among the three pathologies. Concomitant initiation of broad therapy to cover CNS infections has been used in about 35% of patients empirically, at admission^8^. Anti-rickettsia therapy in the first days of the disease contributes to reducing mortality and should not be postponed pending laboratory confirmation^1,3^. The most effective antibiotics are tetracycline derivatives, mainly doxycycline, and treatment should be continued for 7 to 10 days in cases of neurological involvement^3^.

In India, a study of rickettsial infections showed that 88% of children with neurological disease showed good response to the therapy with complete recovery of neurological manifestations, as we observed in our patient. Neurological sequelae such as hemiplegia, deafness, impaired vision, speech disorders, and mental confusion have been described in few cases and may persist for few weeks after treatment^1,4,6^.

## CONCLUSION

The neurological presentation of BSF in children faces a great challenge in systematically integrating rickettsiosis in the differential diagnosis of CNS infections in children, who present febrile and exanthematous conditions^1,4^. The presence of suggestive clinical symptoms associated with the presence of epidemiological data which may favor contact with the tick, should serve as a warning for the diagnosis of the disease in these patients.
